# The role of Y132‐ in Tyrosine‐Kinase dependent suppression of homomeric Kv1.2: Implications for Alzheimer's Disease

**DOI:** 10.1002/alz70855_107170

**Published:** 2025-12-24

**Authors:** Dina Jamshidi, Jack Hyatt, Sebastian Burch, Logen Yaklin, Kristi DeBoeuf, Joseph Farley

**Affiliations:** ^1^ Indiana University, Bloomington, IN, USA

## Abstract

**Background:**

The roles of Aβ in the pathogenesis of Alzheimer 's disease (AD) include disruption of synaptic communication/function and synaptic plasticity mechanisms thought to underlie learning and memory. Exactly how these abnormal processes arise is incompletely understood, but evidence suggests that dysregulation of intracellular Ca2+ levels is involved in alterations of neuronal excitability, synaptic remodeling, and neurodegeneration in AD. Our lab has focused on the potential involvement of voltage‐gated potassium channels (VGKCs) in these processes, particularly Kv1.x family members as they regulate Ca2+ influx, and their inhibition may lead to synapto‐ and neuro‐toxicity through hyperexcitability and excess glutamate release. We have previously observed rapid and robust Aβ(1‐42)‐suppression (∼50% in 30 min) of macroscopic currents mediated by homomeric Kv1.1, Kv1.2, and heteromeric Kv1.1/1.2 channels. BAPTA‐AM treatment, as well as Cyclosporin A (CsA), resulted in significantly less (∼20%) Aβ‐suppression, implicating Ca2+‐dependent PP2B as critical. Additionally, the broad spectrum PTK inhibitor genistein completely eliminated suppression by Aβ(1‐42). Here we sought to understand the role of tyrosine residue Y132 in Kv1.2 in the suppression by Aβ.

**Method:**

The Y132F mutant was generated from a WT KV1.2‐pGEMHE plasmid using a PCR‐based site‐directed mutagenesis kit (NEB), and an invitro transcription reaction was utilized to generate both mutant and WT cRNA. Stage V/VI Xenopus laevis oocytes were injected with ∼1ng of WT Kv1.2 cRNA or Y132F cRNA. The effects of bath application of 1 μM of Aβ(1‐42) were evaluated using two‐electrode voltage clamp electrophysiology (TEVC), with recordings 24‐48 hrs post‐injection.

**Result:**

Baseline currents were unaffected by the Y132F mutation in kinetics or voltage‐dependency compared to WT controls. Similarly, neither WT nor mutant channels significantly differed in their response to 0.01% DMSO with no more than ∼5% suppression observed (*n* = 15, *p* >0.05). However, Y132F exhibited greater resistance to suppression by Aβ(1‐42), with 17% suppression (*n* = 10) compared to the WT 50% 30 minutes post exposure (*n* = 10, *p* < 0.007).

**Conclusion:**

Phosphorylation at site Y132 may be a critical mediator of Aβ based suppression of Kv1.2 current, particularly through endocytotic methods. Suppression of these channels may lead to increased excitotoxicity via increased Ca2+ influx into excitatory neurons and glutamate release.

